# Biosynthesis of acetylacetone inspired by its biodegradation

**DOI:** 10.1186/s13068-020-01725-9

**Published:** 2020-05-15

**Authors:** Yifei Zhou, Yamei Ding, Wenjie Gao, Jichao Wang, Xiutao Liu, Mo Xian, Xinjun Feng, Guang Zhao

**Affiliations:** 1 CAS Key Laboratory of Biobased Materials, Qingdao Institute of Bioenergy and Bioprocess Technology, Chinese Academy of Sciences, Qingdao, 266101 China; 2 University of Chinese Academy of Sciences, Beijing, 100049 China; 3 Institute of Oceanology, Chinese Academy of Sciences, Qingdao, 266071 China; 4 State Key Laboratory of Microbial Technology, Shandong University, Qingdao, 266237 China

**Keywords:** Acetylacetone biosynthesis, Acetylacetone-cleaving enzyme, Rational design, Site-directed mutagenesis, β-Dicarbonyl compounds

## Abstract

**Background:**

Acetylacetone is a commercially bulk chemical with diverse applications. However, the traditional manufacturing methods suffer from many drawbacks such as multiple steps, harsh conditions, low yield, and environmental problems, which hamper further applications of petrochemical-based acetylacetone. Compared to conventional chemical methods, biosynthetic methods possess advantages such as being eco-friendly, and having mild conditions, high selectivity and low potential costs. It is urgent to develop biosynthetic route for acetylacetone to avoid the present problems.

**Results:**

The biosynthetic pathway of acetylacetone was constructed by reversing its biodegradation route, and the acetylacetone was successfully produced by engineered *Escherichia coli* (*E. coli*) by overexpression of acetylacetone-cleaving enzyme (Dke1) from *Acinetobacter johnsonii*. Several promising amino acid residues were selected for enzyme improvement based on sequence alignment and structure analysis, and the acetylacetone production was improved by site-directed mutagenesis of Dke1. The double-mutant (K15Q/A60D) strain presented the highest acetylacetone-producing capacity which is 3.6-fold higher than that of the wild-type protein. Finally, the strain accumulated 556.3 ± 15.2 mg/L acetylacetone in fed-batch fermentation under anaerobic conditions.

**Conclusions:**

This study presents the first intuitive biosynthetic pathway for acetylacetone inspired by its biodegradation, and shows the potential for large-scale production.

## Background

Acetylacetone, also known as 2,4-pentanedione (CAS No. 123-54-6), is an important commodity chemical and widely used as a fuel additive, as dyeing intermediate, in the fields of metal extraction, metal plating, and resin modification [1]. Traditionally, acetylacetone is manufactured through chemical routes using acetone and ketene, which is produced by pyrolysis of acetone or acetic acid at a temperature of 700–800 °C, with carbon monoxide, methane, hydrogen formed as by-products [2, 3]. In specific, esterification of ketene and acetone forms isopropenyl acetate (IPA), in the presence of a strong acid catalyst. Then, IPA is transformed into acetylacetone at 500–600 °C with metallic molybdenum as a catalyst, whereby the yield is only about 45%. In conclusion, the chemical routes suffer from drawbacks such as multiple steps, harsh conditions, low yield, and environmental problems, which hamper further applications of petrochemical-based acetylacetone. To address the issue, new methods need to be developed for acetylacetone preparation. Compared to conventional chemical methods, biosynthetic methods are expected to have advantages such as being eco-friendly and having mild conditions, high selectivity and low potential costs [4], and have been used to produce numerous products, e.g., bio-based chemicals [5], pharmaceuticals [6], biopolymers [7]. For acetylacetone, the theoretical yield is as high as 1.5 mol/mol glucose by bioconversion. Predictably, low cost will also be obtained thanks to the cheap carbon source and the high yield.

Limited studies indicate that acetylacetone is biodegradable [8]. A strain of *Acinetobacter johnsonii* was isolated and proved to have the ability to utilize acetylacetone as a sole carbon source. To reveal the decomposition mechanism, a novel C–C bond-cleaving enzyme, acetylacetone-cleaving enzyme (Dke1, EC 1.13.11.50), was found and purified from *A. johnsonii* [9]. The Dke1 enzyme can activate oxygen to cleave acetylacetone into acetate and methylglyoxal, followed by the conversion of methylglyoxal into lactate by glyoxalase. However, the natural biosynthesis of acetylacetone has not yet been reported. In previous studies, some non-natural products had been bio-synthesized by reversing their biodegradation pathway. An artificial biosynthetic pathway to methylacetoin was constructed by redirecting the methylacetoin biodegradation, and the titer achieved at 3.4 g/L by enzyme screening and metabolic engineering [4]. The direct biocatalytic route of 1,4-butanediol mirroring its biodegradation pathway was reported with a production of 18 g/L [10]. In addition, the engineered reversal of the β-oxidation cycle was adopted for fatty-acid-derived compounds’ biosynthesis, and a series of short-, medium-, and long-chain products were obtained at high yields [11–13]. These successful cases provided a possible strategy to our research for acetylacetone biosynthesis.

**Fig. 1:**
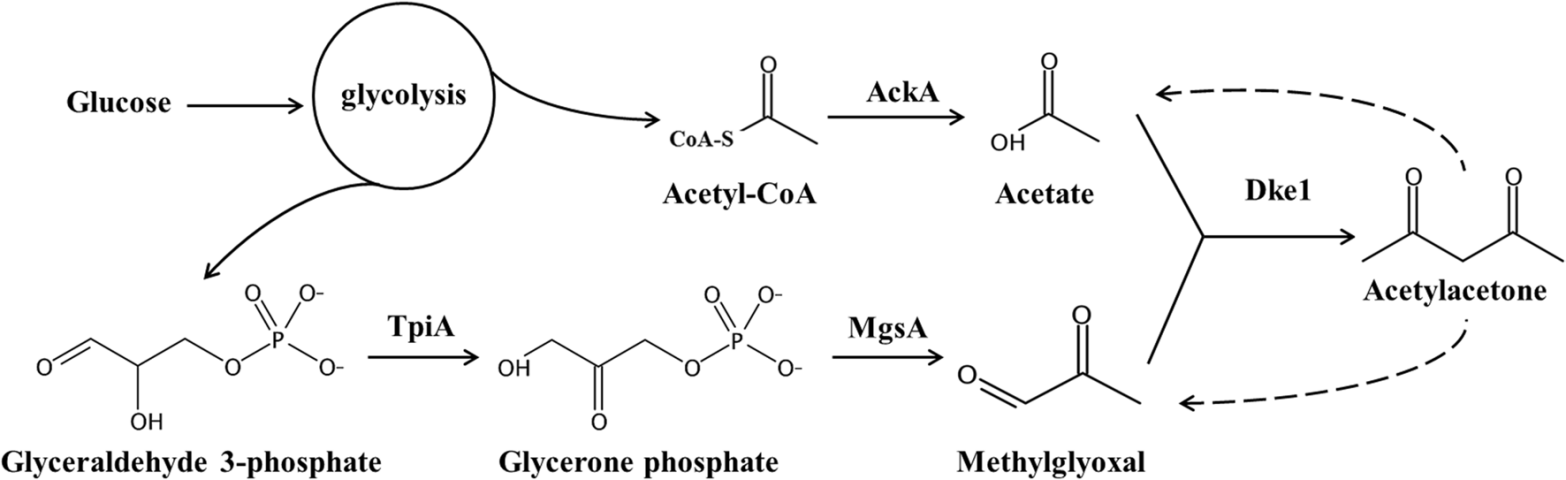
Biodegradation-inspired biosynthetic pathway of acetylacetone. Acetylacetone biodegradation is presented with a dashed line, and the constructed biosynthetic pathways are presented using a solid line. The enzymes used are as follows: AckA, acetate kinase; TpiA, triose-phosphate isomerase; MgsA, methylglyoxal synthase; Dke1, acetylacetone-cleaving enzyme

In this work, we established for the first time the biosynthetic pathway of acetylacetone from fermentable sugars (Fig. 1) inspired by the known acetylacetone biodegradation pathway. It was proved that the acetylacetone decomposition process catalyzed by Dke1 was reversible. The Dke1 activity was improved by rational design, resulting in enhanced acetylacetone production under shake-flask conditions, and the underlying mechanism was proposed. Fed-batch fermentation was conducted to evaluate the potential for large-scale production.

## Results and discussion

### Design and verification of the acetylacetone biosynthesis pathway

Acetylacetone is not a naturally occurring metabolite; however, it can be catabolized by *Acinetobacter johnsonii* as a carbon source [8]. As reported, one molecule of acetylacetone degrades into one acetate and one methylglyoxal catalyzed by acetylacetone-cleaving enzyme Dke1. Methylglyoxal can be produced marginally through glycolysis [14], and acetate is a major product during sugar fermentation especially under anaerobic conditions. Thus, we proposed to achieve acetylacetone production from methylglyoxal and acetate by redirecting the reaction. As shown in Fig. 1, glyceraldehyde 3-phosphate from glycolysis pathway was converted into glycerone phosphate and methylglyoxal by the action of triose-phosphate isomerase (TpiA) and methylglyoxal synthase (MgsA), respectively. Acetyl-CoA was converted into acetate by acetate kinase. Methylglyoxal and acetate were finally transformed into acetylacetone by Dke1.

**Fig. 2:**
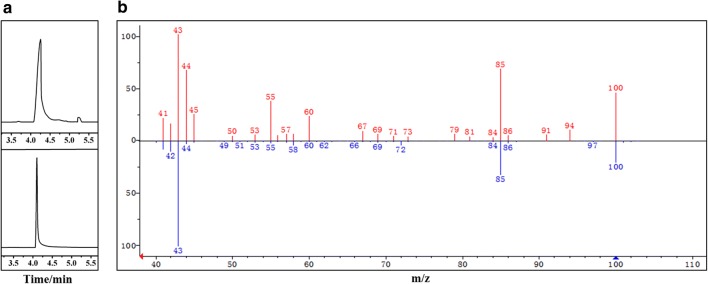
Verification of the production of acetylacetone by engineered *E. coli* strain using GC–MS. GC chromatography (**a**) and mass spectrometry (**b**) results are shown for acetylacetone standard (lower panel) and Q3030 culture (upper panel)

To test the hypothetical acetylacetone biosynthetic pathway, acetylacetone-cleaving enzyme (Dke1) from *A. johnsonii* (accession No. Q8GNT2.1) was cloned into plasmid pETDuet-1, and then the recombinant plasmid was introduced into *E. coli* BL21 (DE3) to construct an acetylacetone producing strain. The expression of the Dke1 was first checked by SDS-PAGE, and the corresponding bands with molecular weights of 17 kDa were observed clearly (Additional file 1: Fig. S1). The resulting strain Q3030 was cultured under shake-flask conditions, and the fermentation products were analyzed by GC–MS. However, no acetylacetone was detected. We then referred back to the acetylacetone biodegradation pathway. As the biodegradation of acetylacetone was activated by oxygen, we speculated the biosynthesis direction was suppressed under aerobic conditions. Thus, anaerobic cultivation in a sealed serum bottle with nitrogen was carried out for further verification. This time, the production of acetylacetone was confirmed by GC–MS analysis. As shown in Fig. 2, a specific peak with a mass of 100 Da was detected, and dissociation of this ion led to other masses such as 43 and 85 Da, exactly the same with acetylacetone standard. Under this condition, the concentration of acetylacetone in fermentation broth was 32.6 ± 1.0 mg/L. Furthermore, the production of acetylacetone was also performed in vitro in Tris buffer (pH 7.5) containing purified Dke1 protein, methylglyoxal and ammonium acetate at 37 °C, and 129.5 ± 8.9 mg/L acetylacetone was accumulated in 24 h. Compared with in vivo system, higher product titer was achieved with in vitro system, probably owing to a relatively simple pathway without side reactions [15].

A sufficient supply of precursors is necessary for the efficient synthesis of the final products [16]. As one of the main metabolites in *E. coli* under anaerobic condition, acetate should be sufficient for acetylacetone biosynthesis. But in general, methylglyoxal synthesis is inhibited in cell [14, 17]. So the methylglyoxal content in the flask was improved by extra supplement with a concentration of 0.1 mM or overexpression of the key genes (*tpiA* and *mgsA*) for enhanced acetylacetone production (Fig. 1). However, no more acetylacetone was accumulated. Meanwhile, the in vitro experiment with 20 mM methylglyoxal was conducted, and the production of acetylacetone (128.9 ± 8.6 mg/L) did not show significant difference with that with 10 mM methylglyoxal (*p* = 0.94). So, we speculated that low activity of Dke1 might be the primary reason of low acetylacetone yield. Then, the acetylacetone-cleaving enzyme was first screened. Two more genes encoding acetylacetone-cleaving enzyme from *Paraburkholderia xenovorans LB400* (P_Dke1, accession No. NC_007951.1) and *Tistrella mobilis* (T_Dke1, accession No. NC_017966.1) were cloned and used for acetylacetone production. Unfortunately, no acetylacetone was detected using engineered strains with P_Dke1 or T_Dke1. So, other strategies should be considered for higher acetylacetone production.

### Rational design of Dke1

Protein engineering using directed evolution or rational design has been developed rapidly, and has acted as a powerful tool for enzyme improvement [18, 19]. Especially, protein sequence information combined with computational modeling tools can be used to identify promising amino acid sites and can be selected for enzyme engineering [20]. Thus, rational design strategies were considered for increasing Dke1 activity.

**Fig. 3:**
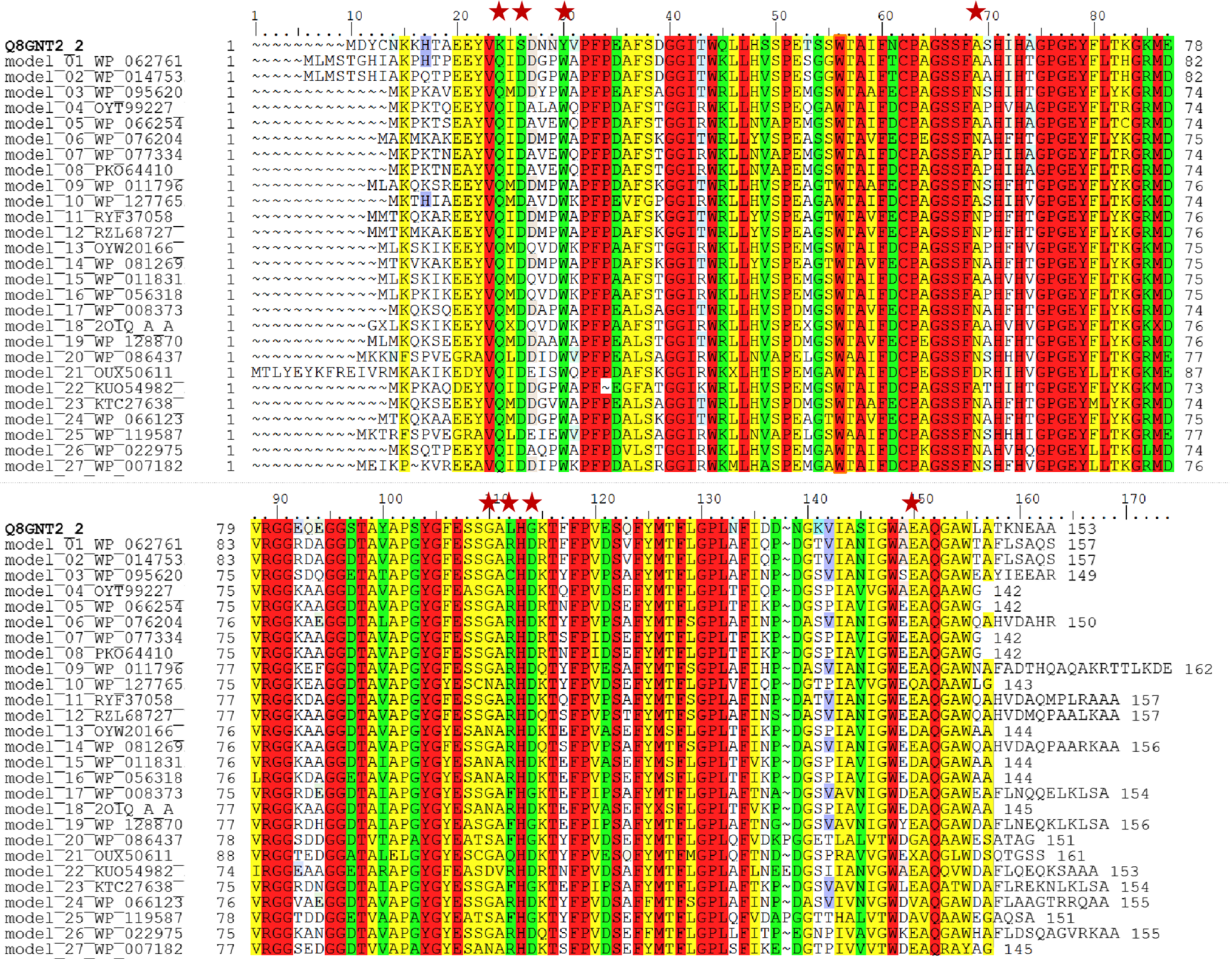
Alignment of multiple amino acid sequences of Dke1 and other proposed acetylacetone-cleaving enzymes. The definitely conserved sites are labeled in red, the relatively conserved sites are labeled in yellow, the variable sites are labeled in green and the selected sites for mutation are marked with red star

**Fig. 4:**
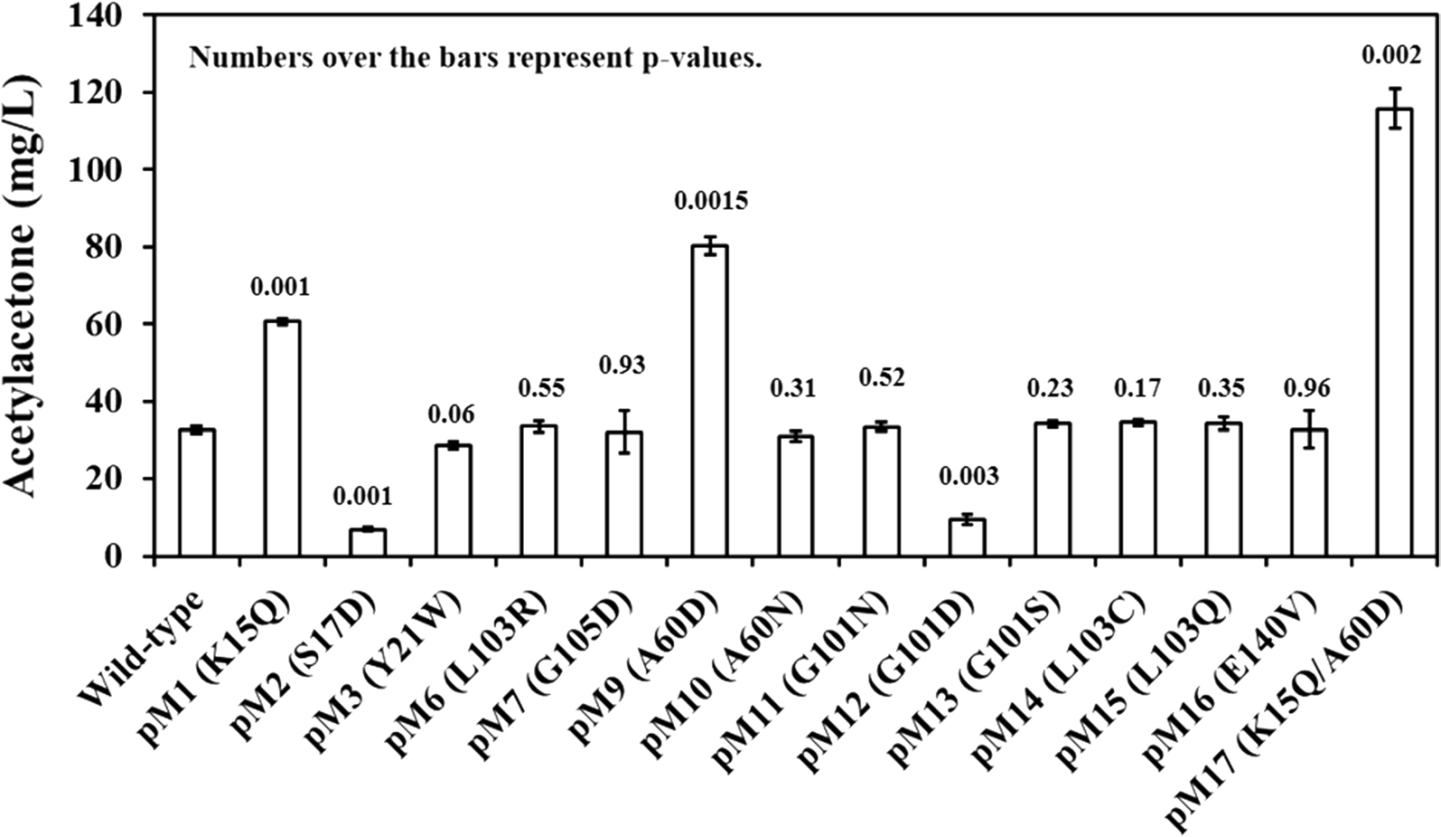
Effect of Dke1 mutagenesis on acetylacetone production in flask cultivation. Data were obtained after each strain was induced for 24 h in liquid LB medium. All the experiments were carried out in triplicate. All *p* values are based on two-tailed tests (wild type and mutant strain) and presented over the bars

To improve the Dke1 catalytic activity, multiple amino acid sequence alignment was performed between Dke1 and 27 other acetylacetone-cleaving enzymes or hypothetical proteins sharing more than 60% identity with Dke1 (Additional file 2: Table S1). Among 153 amino acid residues in Dke1, 52 residues were conserved in all 28 proteins, and referred to as definitely conserved sites (highlighted in red in Fig. 3); 44 amino acid residues appeared in more than 14 other proteins, and defined as relatively conserved sites (highlighted in yellow in Fig. 3); and 19 amino acid residues rarely (less than 26%) showed up in other proteins, and were called variable sites (highlighted in green in Fig. 3). Among the variation sites, three amino acid residues were found conserved in all proteins except Dke1, and site-directed mutagenesis was performed to construct Dke1 single mutants harboring each substitution as its homologs. Then, shake flask cultivation was performed to test the effect of each mutation. The strain with Dke1 K15Q (Q3080) accumulated 60.6 ± 0.7 mg/L acetylacetone, which is 1.9-fold higher than that of the wild-type strain Q3030 (*p* < 0.01) (Fig. 4). However, the strain with Dke1 Y21W (Q3082) only produced 28.8 ± 1.0 mg/L acetylacetone, and the production reduced dramatically to 7.0 ± 0.4 mg/L when Dke1 S17D was used in strain Q3081.

Meanwhile, 3D structure analysis of the wild-type Dke1 was performed based on its crystal structure (PDB ID: 3BAL). In the crystal structure of the ligand-free Dke1, the Fe^2+^-coordinating His-62, His-64 and His-104 comprise the substrate catalytic center [21]. The definitely conserved sites occupy about three quarters of the substrate channel, which are the most important amino acid residues for maintaining structure and function of the proteins (Additional file 1: Fig. S2). These sites would not be modified in this study. Most of the relatively conserved sites cover about one quarter of the substrate channel, while a few sites are located close to the catalytic center. We analyzed the possible impact from the mutation of the relatively conserved sites near the catalytic center and all the variation sites. By analysis of the key influence factors including properties of amino acids, hydrogen bonding, electron distribution, and charge properties, 5 sites were selected as potential mutation sites for improving Dke1 activity (Table 1). For example, the carbonyl oxygen of A60 was interacted with H62 by forming a hydrogen bond [22], and it will transfer electrons to H62 after mutated to D60 or N60 which might be helpful to enhance the catalytic activity. Then, 10 mutants were constructed and compared with Dke1 wild-type protein. As shown in Fig. 4, the strain with Dke1 A60D (Q3148) produced 80.2 ± 2.4 mg/L acetylacetone which is 2.5-fold higher than that of the strain Q3030 (*p* < 0.01). The strain with Dke1 G101D (Q3151) only produced 9.5 ± 1.4 mg/L acetylacetone while the ability of other mutants was basically the same with wild-type Dke1 protein (*p* > 0.1).

**Table 1:** Analysis of the proposed sites for site-directed mutagenesis

Proposed sites	Mutation information	Annotation
A60	N60 or D60	The carbonyl oxygen of A60 (Additional file 1: Fig. S3b) is hydrogen bonded to H62. The electrons could be transferred to H62 after Ala mutated to Asp or Asn
G101	N101, D101 or S101	The N of the main chain of G101 (Additional file 1: Fig. S3c) is hydrogen bonded to the O of the main chain of H64. When Gly was mutated to Asp, Asn or Ser, the electron distribution of site 101 will change and thus affect the catalytic activity
L103	R103, Q103 or C103	L103 (Additional file 1: Fig. S3c) was located close to H104; the property of site 103 directly affects H104. When the nonpolar amino acid (Leu) was mutated to polar amino acid (Gln, Cys) or alkaline amino acid (Arg), the influence to H104 from site 103 may change and thus affect the catalytic activity
G105	D105	When G105 (Additional file 1: Fig. S3c) was mutated to D105, the electronegativity of the catalytic activity center will be significantly increased
E140	V140	E140 (Additional file 1: Fig. S3d) with hydrophobic amino acids around was located on the outer edge of the hydrophobic channel. When the acidic amino acid (Glu) was mutated to a hydrophobic amino acid (Val), it might be more conducive to discharge the catalytic product

As the mutations K15Q and A60D had shown improvement in acetylacetone production, the influence of the double mutant on acetylacetone production was also assessed. As Fig. 4 demonstrated, the strain carrying Dke1 K15Q/A60D (Q3170) exhibited the highest acetylacetone synthesis efficiency, and 115.7 ± 5.1 mg/L acetylacetone was accumulated in the culture, which was 3.6-fold higher than that of strain Q3030 (*p* < 0.01). Dke1 activity was assayed in vitro using purified proteins and the activity of each enzyme showed a similar trend with acetylacetone production (Table 2).

**Table 2:** Activity of the Dke1 and its mutants (*p *< 0.01 compared with the wild type)

Enzyme	Specific activity (μmol/min/mg protein)
WT	0.8 ± 0.01 × 10^−3^
K15Q	1.7 ± 0.07 × 10^−3^
A60D	2.5 ± 0.02 × 10^−3^
K15Q/A60D	3.0 ± 0.07 × 10^−3^

### Structural simulation and molecular docking of Dke1

**Fig. 5:**
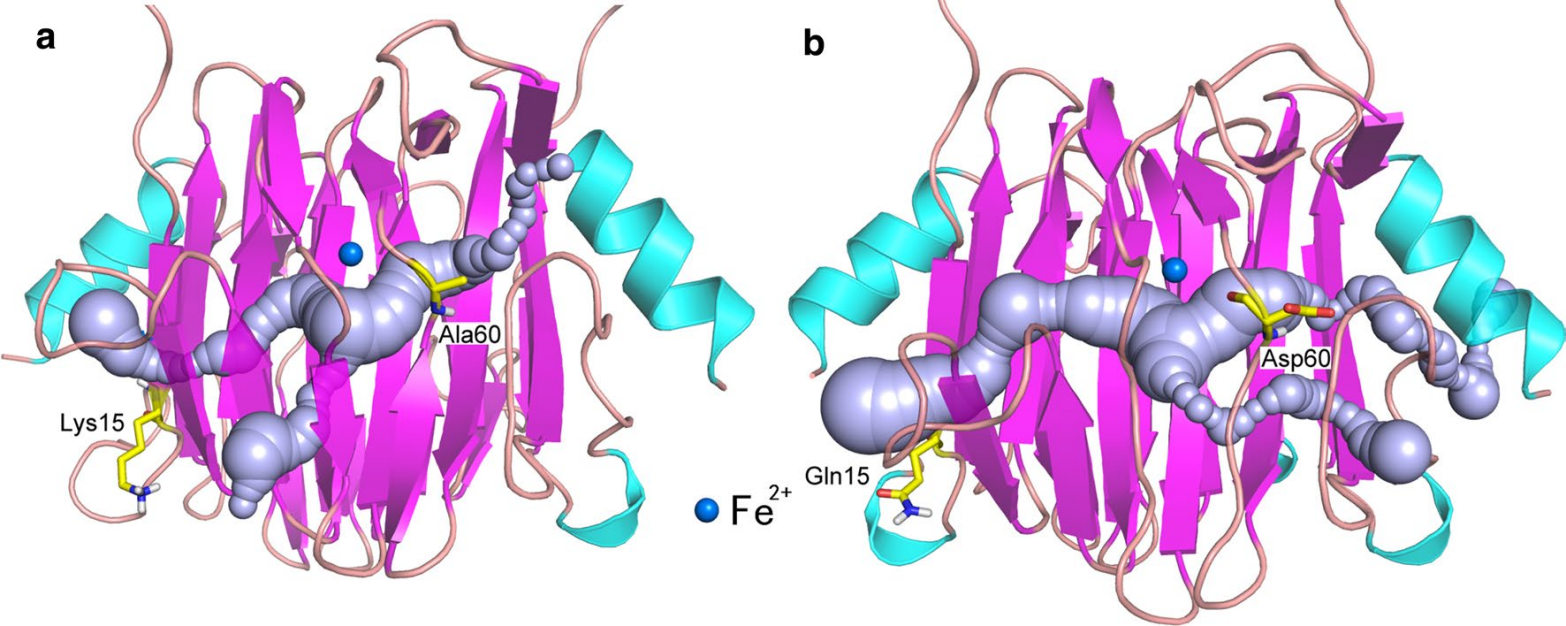
Analysis of the Dke1 structure with molecular docking. **a** Wild-type. **b** K15Q/A60D mutant. The β-pleated sheet shown in purple, the α-helix shown in light blue, and the substrate channel shown in grey

To reveal the molecular basis for increased enzymatic activity of Dke1 (K15Q/A60D), in silico structural modeling and molecular docking were performed using the 3D structure of Dke1 (PDB ID: 3BAL) as a model template. As shown in Fig. 5, no remarkable differences in the overall structure have been observed between wild-type and mutated Dke1 protein, implying that the variant is correctly folded. Interestingly, with the mutation of K15Q, the channel, through which substrates enter the enzyme reactive center, was widened. Furthermore, a neutral residue glutamine is less attractive to negative-charged acetate than lysine which is positive charged. Taken together both factors make it easier for acetate to arrive at the reactive site of Dke1. The residue A60, as a hydrophobic amino acid, is located close to the reactive center and its side chain is oriented toward the interior of the protein. The substitution of alanine by a hydrophilic residue, aspartate, changed the orientation of the side chain at position 60, resulting in a reactive center with increased volume, which may be more conducive for substrate binding. In summary, these changes brought about by the double mutant increased substrate access to the enzyme active site, resulting in enhanced enzyme activity.

### Fed-batch fermentation

**Fig. 6:**
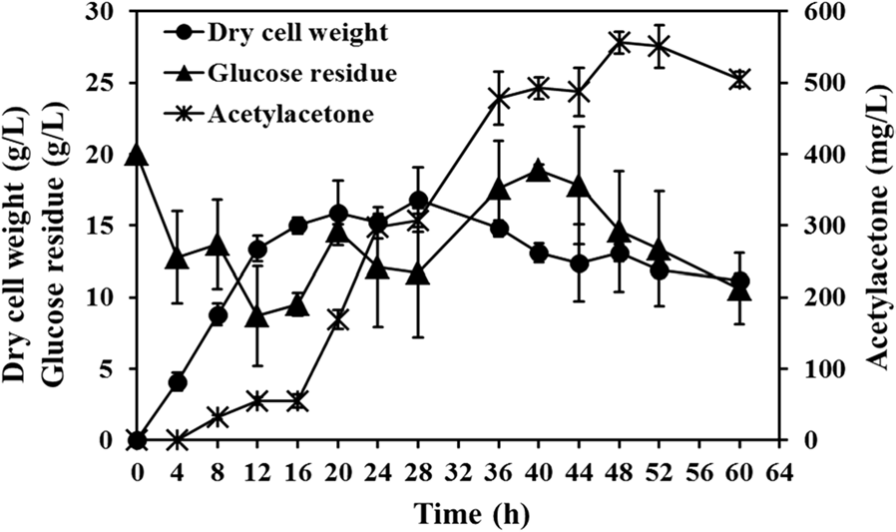
The time profiles of cell growth, residual glucose and acetylacetone accumulation in fed-batch fermentation performed in a 5-L laboratory bioreactor. Dry cell weight was marked with circle, residual glucose was marked with triangle, and acetylacetone was marked with asterisk

As the strain Q3170 presented the highest acetylacetone production in flask cultivation, fed-batch fermentation was conducted in a 5-L bioreactor. The concentrated glucose was used for cell growth and controlled between 10 and 20 g/L during fermentation. 0.2 mM IPTG was added to induce the recombinant proteins when the cell density reached to about 30 OD_600_ at 12 h. Simultaneously, the sterilized air was switched to high-purity nitrogen for anaerobic environments. Cell growth, residual glucose and acetylacetone accumulation were monitored over the course of the fermentation. As shown in Fig. 6, the cell mass reached to a maximum 16.8 g DCW/L at 28 h and decreased slightly after 36 h. A small amount of acetylacetone had already been synthesized before induction possibly due to Dke1 basal expression. After induction, acetylacetone rapidly accumulated and reached the maximum of 556.3 ± 15.2 mg/L at 48 h with a yield of 5.1 mmol/mol glucose. The productivity of 21.2 mg/L/h was achieved during 16–36 h while only 1.1 mg/L/h during the latter 24 h.

Although the yield of acetylacetone from recombinant *E. coli* in this study was still relatively low, *E. coli* is a competitive species for industrial applications with short multiplication time, growth with inexpensive carbon sources and amenability to genetic modification [23]. Many strategies can be used for improving the acetylacetone yield in future study, such as metabolic flow regulation [24] and key genes expression level modulation [25]. Previous study has shown that acetylacetone has toxic side effects on the immune system of mammals [26], various aquatic organisms [27] and microorganisms [28]. The toxicity threshold of acetylacetone is 67 mg/L for *Pseudomonas putida* [28], and an excess of acetylacetone (> 1.5 g/L) can completely inhibit the cell growth of *A. johnsonii* [9]. We speculated that the acetylacetone toxicity might be one of the main reasons for the low yield. The toxicity of acetylacetone to *E. coli* cells was tested (Additional file 1: Fig. S4). The results showed that 100 mg/L acetylacetone has obvious inhibition effect on the growth of *E. coli*, and the cell was almost completely inhibited at a concentration of 5 g/L. The mechanism of toxicity, however, has still not been revealed. Further research is needed on the underlying mechanism to guide the metabolic engineering for higher production. Adaptive evolution is also an effective measure to improve the bacterial tolerance and enhance the production [29]. As *E. coli* naturally produces a mixture of ethanol, hydrogen, organic acids, such as lactic acid, succinic acid, acetic acid under anaerobic conditions [30], metabolic flow analysis and regulation are necessary to conduct for acetylacetone production enhancement. In addition, as the cultivation conditions play an important role on cell growth, product synthesis, and conversion efficiency, it is also worthy to optimize the medium components, substrate addition strategy, fermentation mode, etc. in future research.

## Conclusions

The biosynthetic pathway of acetylacetone was constructed, and the acetylacetone was produced successfully from glucose by engineered *E. coli* by overexpression of acetylacetone-cleaving enzyme (Dke1). The production was improved by site-directed mutagenesis of Dke1 and the double mutant (K15Q/A60D) enabled the highest acetylacetone-producing capacity. Finally, 556.3 ± 15.2 mg/L acetylacetone was obtained at 36 h post-induction in fed-batch fermentation under anaerobic condition. This study presents the first intuitive biosynthetic pathway of acetylacetone inspired by its biodegradation, and shows its potential for large-scale production. As reported, Dke1 is not absolutely specific for acetylacetone. Many related β-dicarbonyl compounds such as 3,5-heptanedione, 2,4-octanedione, 2-acetylcyclohexanone, ethylacetoacetate, etc. can be accepted as substrate by Dke1 [8, 9]. As a consequence, the results of this study also provide a possible biosynthesis method for other β-dicarbonyl compounds.

## Materials and methods

### Strains and plasmids

All strains and plasmids used in this study are listed in Table 3. The primers used for plasmid construction and allele verification are listed in Additional file 2: Table S2. *E. coli* DH5α (Invitrogen) was used for plasmids’ preparation and BL21 (DE3) was used for recombinant protein expression and acetylacetone production. The genes encoding acetylacetone-cleaving enzyme from *A. johnsonii*, *Paraburkholderia xenovorans LB400*, and *Tistrella mobilis* were codon optimized and chemically synthesized by Beijing Liuhe BGI, and cloned into pETDuet-1 between EcoRI and BamHI sites to construct plasmids pETDuet-Dke1, pETDuet-P_Dke1, and pETDuet-T_Dke1, respectively. Mutations were introduced into the Dke1 gene in pETDuet-Dke1 using an overlap extension PCR method [31]. The resulting plasmids were named pM1 to pM19, respectively. All the recombinant plasmids were verified by colony PCR and nucleotide sequencing.

**Table 3:** Strains and plasmids used in this study

Strains or plasmids	Genotype/description	Source
Strains		
*E. coli* DH5	F^−^*rec*A *endA1 Φ80dlacZΔM15 hsdR17*(*r*_*K*_^−^*m*_*K*_^+^)*λ*^−^	Invitrogen
*E. coli* BL21 (DE3)	F^−^*ompT hsdS*_*B*_*(r*_*B*_^−^*m*_*B*_^−^*) gal dcm* λ (DE3)	Invitrogen
Q2837	*E. coli* BL21(DE3) carrying pETDuet-Dke1 and pACYCDuet-MgsA-TpiA	This study
Q3028	*E. coli* BL21(DE3) carrying pETDuet-Tmo_Dke1	This study
Q3029	*E. coli* BL21(DE3) carrying pETDuet-Pxe_Dke1	This study
Q3030	*E. coli* BL21(DE3) carrying pETDuet-Dke1	This study
Q3080	*E. coli* BL21(DE3) carrying pM1	This study
Q3081	*E. coli* BL21(DE3) carrying pM2	This study
Q3082	*E. coli* BL21(DE3) carrying pM3	This study
Q3085	*E. coli* BL21(DE3) carrying pM6	This study
Q3086	*E. coli* BL21(DE3) carrying pM7	This study
Q3148	*E. coli* BL21(DE3) carrying pM9	This study
Q3149	*E. coli* BL21(DE3) carrying pM10	This study
Q3150	*E.coli* BL21(DE3) carrying pM11	This study
Q3151	*E. coli* BL21(DE3) carrying pM12	This study
Q3152	*E. coli* BL21(DE3) carrying pM13	This study
Q3153	*E. coli* BL21(DE3) carrying pM14	This study
Q3154	*E. coli* BL21(DE3) carrying pM15	This study
Q3155	*E. coli* BL21(DE3) carrying pM16	This study
Q3170	*E. coli* BL21(DE3) carrying pM1*7*	This study
Plasmids		
pETDuet-1	Amp^R^, rep_pBR322_, *lacI* P_T7_	Novagen
pACYCDuet-1	Cm^R^, p15A origin, lacI P_T7_	Novagen
pETDuet-Dke1	pETDuet-1 harboring acetylacetone-cleaving enzyme (Dke1) from *A. johnsonii*	This study
pETDuet-Tmo_Dke1	pETDuet-1 harboring acetylacetone-cleaving enzyme from *T. mobilis*	This study
pETDuet-Pxe_Dke1	pETDuet-1 harboring acetylacetone-cleaving enzyme from *P. xenovorans*	This study
pACYCDuet-MgsA-TpiA	pACYCDuet-1 harboring methylglyoxal synthase (MgsA) and triose-phosphate isomerase (TpiA) from *E. coli*	This study
pM1	rep_pBR322_ Amp^R^*lacI* P_T7_ Dke1^K15Q^	This study
pM2	rep_pBR322_ Amp^R^*lacI* P_T7_ Dke1^S17D^	This study
pM3	rep_pBR322_ Amp^R^*lacI* P_T7_ Dke1^Y21W^	This study
pM6	rep_pBR322_ Amp^R^*lacI* P_T7_ Dke1^L103R^	This study
pM7	rep_pBR322_ Amp^R^*lacI* P_T7_ Dke1^G105D^	This study
pM9	rep_pBR322_ Amp^R^*lacI* P_T7_ Dke1^A60D^	This study
pM10	rep_pBR322_ Amp^R^*lacI* P_T7_ Dke1^A60N^	This study
pM11	rep_pBR322_ Amp^R^*lacI* P_T7_ Dke1^G101N^	This study
pM12	rep_pBR322_ Amp^R^*lacI* P_T7_ Dke1^G101D^	This study
pM13	rep_pBR322_ Amp^R^*lacI* P_T7_ Dke1^G101S^	This study
pM14	rep_pBR322_ Amp^R^*lacI* P_T7_ Dke1^L103C^	This study
pM15	rep_pBR322_ Amp^R^*lacI* P_T7_ Dke1^L103Q^	This study
pM16	rep_pBR322_ Amp^R^*lacI* P_T7_ Dke1^E140V^	This study
pM17	rep_pBR322_ Amp^R^*lacI* P_T7_ Dke1^K15Q/A60D^	This study

### Protein expression and gel electrophoresis analysis

To check the expression of the recombinant proteins, single colonies of *E. coli* BL21 (DE3) harboring different recombinant plasmids were cultured in LB medium containing appropriate antibiotics at 37 °C overnight and then diluted 1:100 into fresh LB medium and induced with 0.2 mM isopropyl-β-d-thiogalactopyranoside (IPTG) at an OD_600_ of 0.6–0.8. The cells were collected from 10 mL bacteria cultures 4 h post-induction and washed with phosphate buffer (pH 6.8). The washed cells were suspended in 1 mL buffer and subjected to ultra-sonication (SCIENTZ JY92-IIN, 300 W, 3 s pulse on and 3 s pulse off for 5 min). The cell lysates were centrifuged and the protein expression was analyzed by SDS-PAGE.

### Dke1 activity assay

The in vitro reaction system (1 mL) for the activity assay of Dke1 contained 0.1 μM purified enzyme, 0.5 mM FeSO_4_·7H_2_O, 10 mM MgCl_2_·6H_2_O, 10 mM KCl, 1 mM DTT, 10 mM methylglyoxal and 10 mM ammonium acetate in 20 mM Tris/HCl buffer (pH 7.5) and was incubated at 37 °C for 24 h. The reaction mixture was centrifuged at 5000×*g* for 5 min, and then the supernatant was subjected to HPLC or GC–MS analysis to verify acetylacetone production.

### Shake-flask cultivation

To evaluate the acetylacetone production using different engineered strains, shake-flask cultivations were carried out with 50 mL of liquid LB medium containing 20 g/L glucose in 250-mL non-baffled flasks or serum bottles with appropriate antibiotics. When necessary, ampicillin and chloramphenicol were added at a final concentration of 100 μg/mL and 50 μg/mL, respectively. The strains were inoculated to the medium and incubated in an orbital shaker incubator at 37 °C and 180 rpm. 0.2 mM IPTG was added into the medium to induce the recombinant protein expression when the cells reached at about 0.6–0.8 OD_600_. Nitrogen protection was introduced to create an anaerobic environment in the serum bottle. After induction, the temperature was set at 30 °C for further 24 h cultivation. All shake-flask experiments were performed in triplicate.

### Fed-batch fermentation

For large-scale production, fed-batch fermentation was carried out in a Biostat B plus MO5L bioreactor (Sartorius Stedim Biotech GmbH, Germany) containing 2 L growth medium (20 g/L tryptone, 10 g/L yeast extract, 20 g/L NaCl, 3 g/L KH_2_PO_4_, 0.26 g/L MgSO_4_, 1.0 g/L NH_4_Cl, 15.2 g/L Na_2_HPO_4_, 20 g/L glucose and 2 mL of trace element solution). The trace element solution contained (per liter) 3.7 g (NH_4_)_6_Mo_7_O_24_·4H_2_O, 2.47 g H_3_BO_3_, 1.58 g MnCl_2_·4H_2_O, 0.29 g ZnSO_4_·7H_2_O, and 0.25 g CuSO_4_·5H_2_O. Two hundred milliliters of overnight seed culture was inoculated into the bioreactor to start the fermentation at 37 °C. During the fermentation, sterilized air was supplied at 1 vvm and ammonia was added automatically to control the pH 7. The agitation speed was set at 400 rpm and then associated with the dissolved oxygen to maintain the concentration at 20% saturation. Fed-batch mode was commenced by feeding 60% glucose when the dissolved oxygen increased. When the cell density reached to an OD_600_ of 30, the recombinant proteins were induced by 0.2 mM IPTG along with 0.5 mM FeSO_4_·7H_2_O added, and nitrogen was used for anaerobic conditioning after induction. The temperature was adjusted to 30 °C for further cultivation. The agitation speed was kept at a constant rate of 200 rpm during anaerobic fermentation. More details of the fed-batch fermentation are presented in Additional file 1: Fig. S5 and Additional file 2: Fed-batch fermentation. 5 mL of the fermentation broth was withdrawn periodically to determine the cell density, residual glucose and product titer. The fed-batch fermentation was performed in triplicate.

### Molecular docking

Molecular docking of acetylacetone with Dke1 was carried out with AutoDock 4.2.6 program. The initial structure was prepared using AutoDockTools 1.5.6 [32], preserving the original charge of the protein and generating a pdbqt file for docking. The 3D structure of the acetylacetone was downloaded from the PubChem database. MOPAC program was then used to optimize the structure and calculate the PM3 atomic charge. The structure of acetylacetone was also prepared by AutoDock Tools 1.5.6, and the corresponding pdbqt file was generated for docking. The active site of Dke1 was chosen as the binding pocket for docking. The number of grid points in the XYZ of grid box was set to 40 × 40 × 40, the grid spacing was 0.375 Å, the number of Genetic Algorithm (GA) run was set to 100, and the rest parameters were set to default. Finally, the structure with the lowest docking energy was carried out with energy minimization. The optimization process is carried out in two steps: first, the steepest descent method optimization of 2000 steps, then the structure was further optimized by the 2000 steps with conjugate gradient method.

### Analytic methods

The OD at 600 nm was routinely used to monitor cell growth via ultraviolet spectrophotometer (Varian Cary 50 UV–Vis, US), and one unit of OD_600_ was equivalent to 0.43 g DCW/L [33]. The residual glucose was determined by an SBA-40D biosensor analyzer (Institute of Biology, Shandong Academy of sciences, China). The culture samples were centrifuged at 10,000×*g* for 10 min; the supernatants were filtered through a 0.2-μM Tuffryn membrane (China) and used for HPLC analysis (Waters 1525, 300 mm × 7.8 mm Aminex HPX-87H, UV–Vis detector at 280 nm, for more detailed information see Additional file 1: Fig. S6 and Additional file 2: Standard curve establishment and Table S3). To further confirm acetylacetone production in our cultures, the same sample was analyzed by GC–MS after HPLC determination. The GC–MS analysis was performed with an Agilent GC quadrupole instrument. The analysis conditions were as follows: a 30-m HP-5 column (internal diameter 0.25 mm, film thickness 0.25 μm), the column temperature program was composed of an initial hold at 50 °C for 5 min, ramping at 15 °C per min to 240 °C and holding for 5 min. The injector and transfer line temperature were 240 and 250 °C, respectively. The mass spectrometry full scan was from 30 to 400, the ion source and quadrupole temperature were 230 and 150 °C, respectively.

## Supplementary information

**Additional file 1.** Additional figures.

**Additional file 2.** Additional tables.

## Data Availability

We provide all the necessary data for the publication of this article. All additional data are present in the article and the additional material documents.

## Abbreviations

Dke1: Acetylacetone-cleaving enzyme
TpiA: Triose-phosphate isomerase
MgsA: Methylglyoxal synthase
*E. coli*: *Escherichia coli*
*A. johnsonii*: *Acinetobacter johnsonii*
GC–MS: Gas chromatography–mass spectrometer
HPLC: High-performance liquid chromatography
OD_600_: Optical density at wavelength 600 nm
DTT: Dithiothreitol
IPTG: Isopropyl-β-d-thiogalactoside
DCW: Dry cell weight
