# Supplementary material for: Biosynthesis of acetylacetone inspired by its biodegradation
Source: Biotechnol Biofuels. 2020 May 15;13:88. doi: 10.1186/s13068-020-01725-9 (PMC7226712; doi:10.1186/s13068-020-01725-9)

**Figure captions**

**Figure S1** SDS-PAGE analysis of the expression of different acetylacetone cleaving enzymes. The arrows indicate Dke1~17kDa. Lane M: prestained protein molecular weight maker; Lane 1-2: supernatant and precipitate of *E.coli* BL21(DE3); Lane 3-4: supernatant and precipitate of Q3030; Lane 5-6: supernatant and precipitate of Q3029; Lane 7-8: supernatant and precipitate of Q3028.

**Figure S2** The distribution of different amino acids in 3D structure of Dke1. The definitely conserved region marked with red, the relatively conserved region marked with yellow and the variation sites marked with green. (a): The overall structure of Dke1; (b): The definitely conserved amino acids region; (c): The relatively conserved amino acids region; (d): The variation sites.

**Figure S3** The distribution of the selected main mutation sites. (a): The active center of Dke1; (b): The location of A60; (c): The location of G101, L103 and G105; (d): The location of I136-E140; (e): The location of some other amino acids.

**Figure S4** Growth of *E. coli* BL21(DE3) wild-type strain with acetylacetone at different concentrations.

**Figure S5** The DO-stir rate profile. DO, blue line with circle; stir rate, brown line with square.

**Figure S6** The representative chromatogram of the standard (lower panel) and the sample (upper panel).

Fig. S1


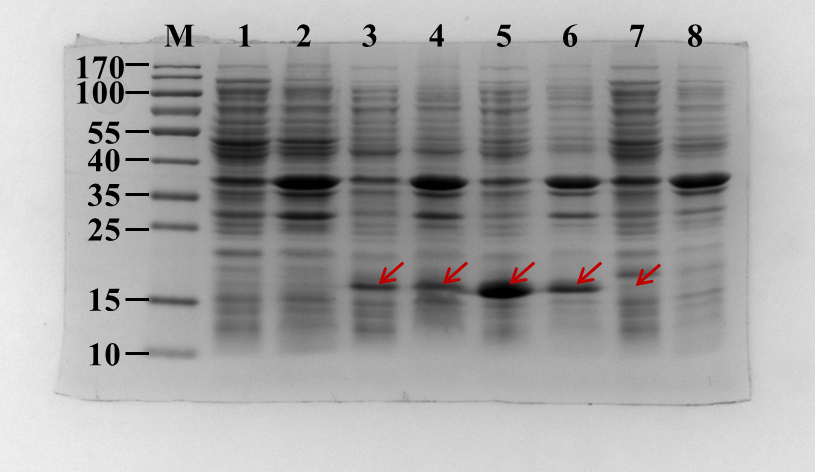


Fig. S2


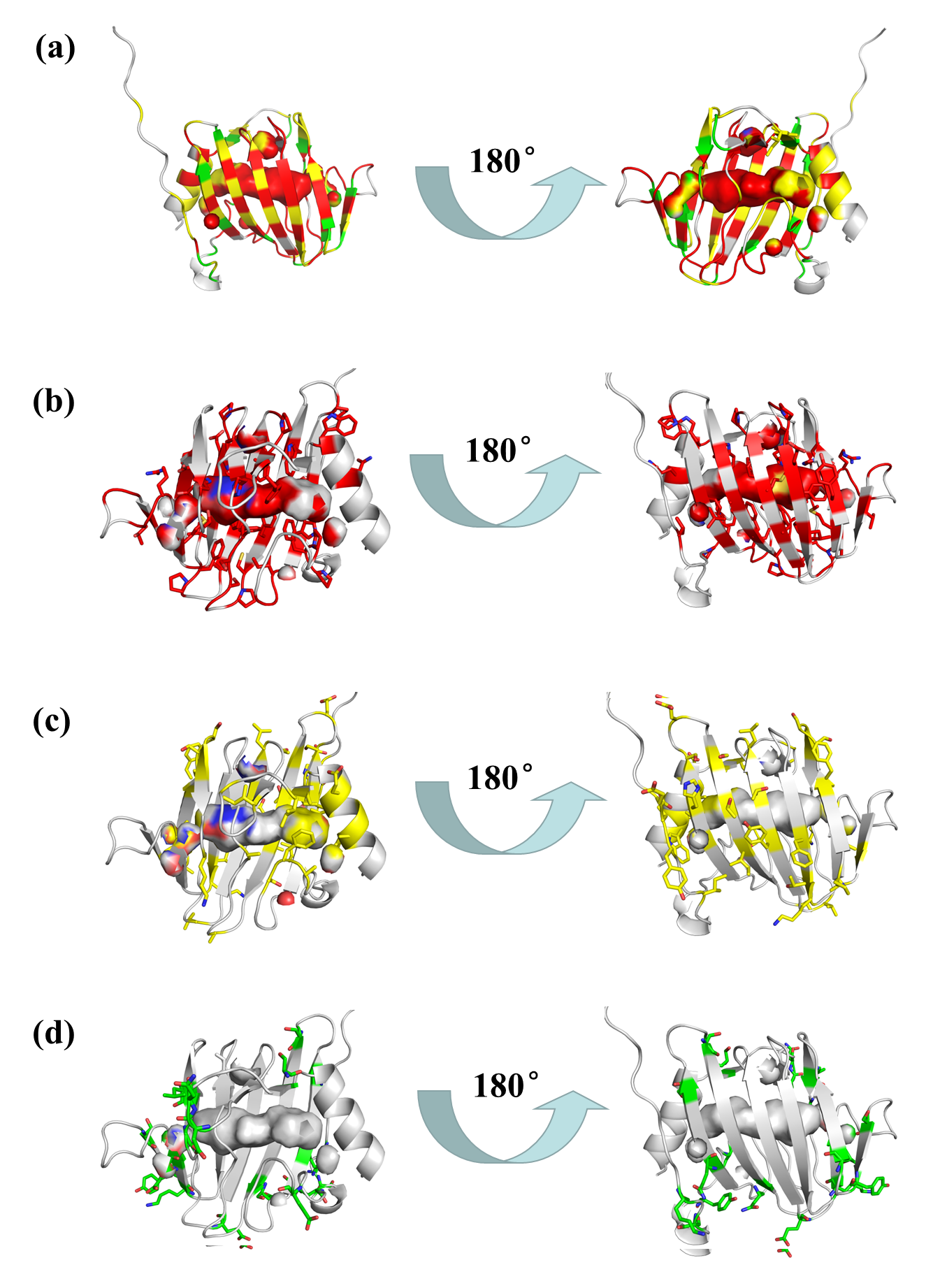


Fig. S3


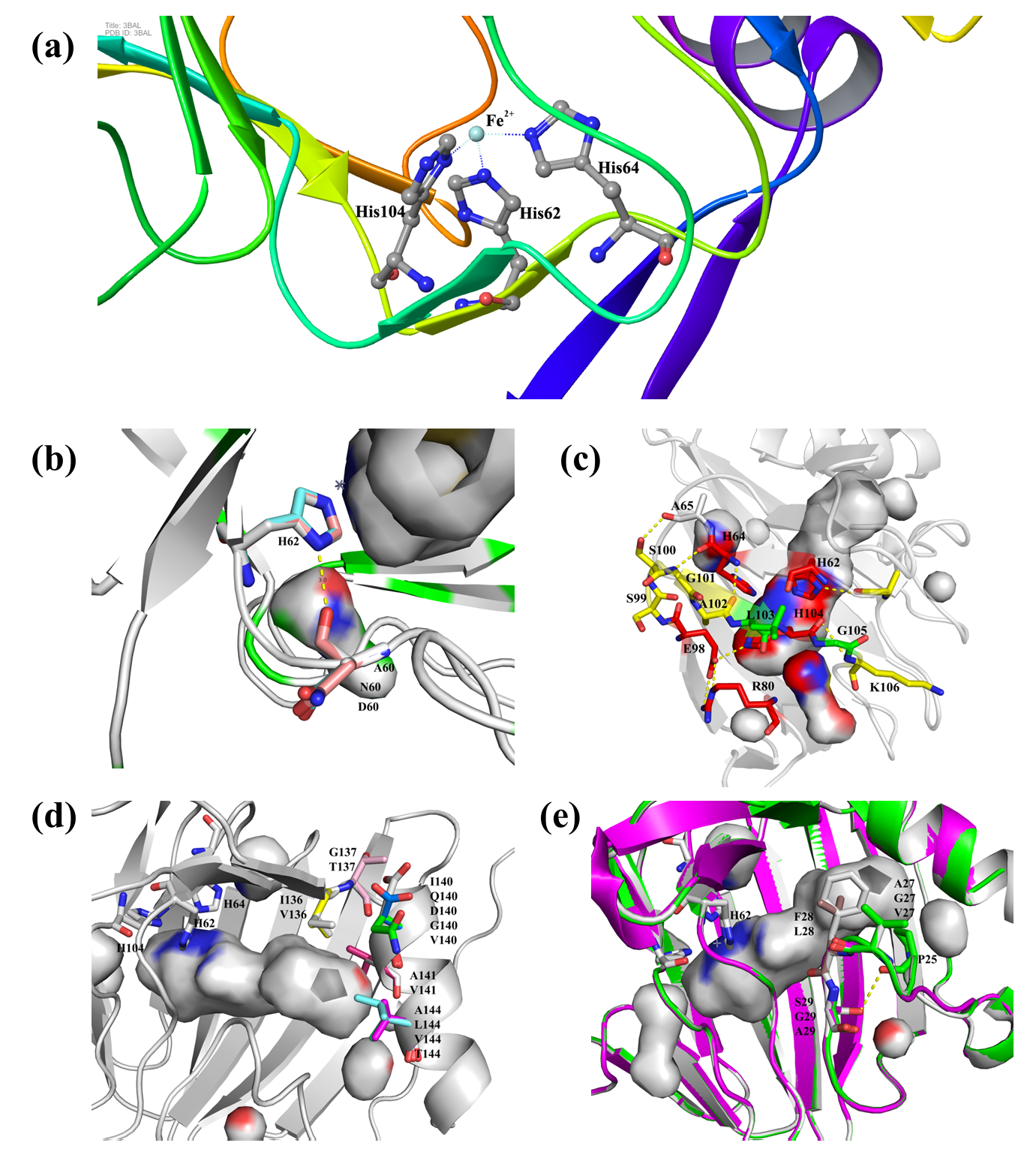


Fig. S4


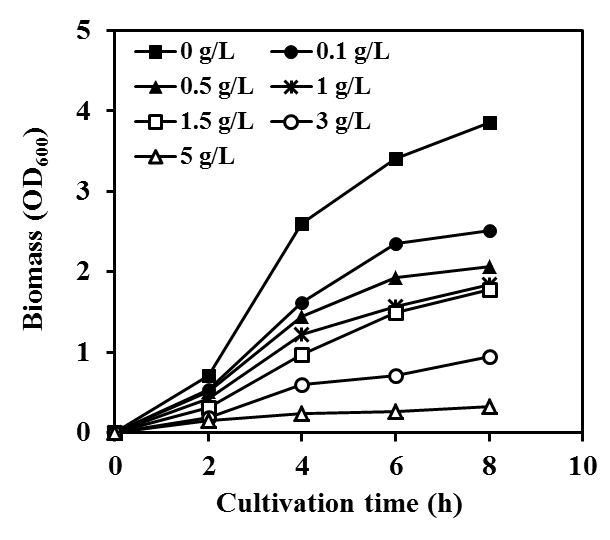


Fig. S5


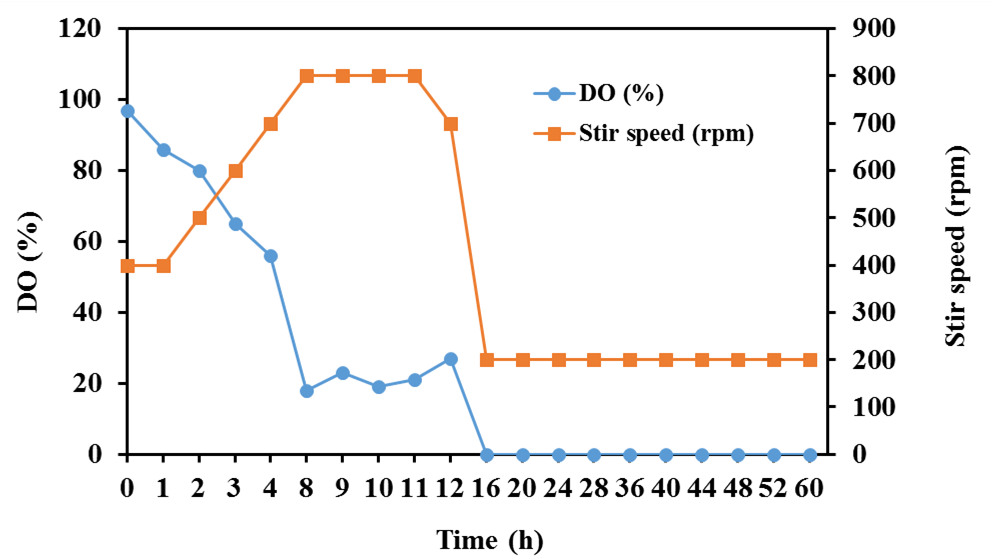


Fig. S6


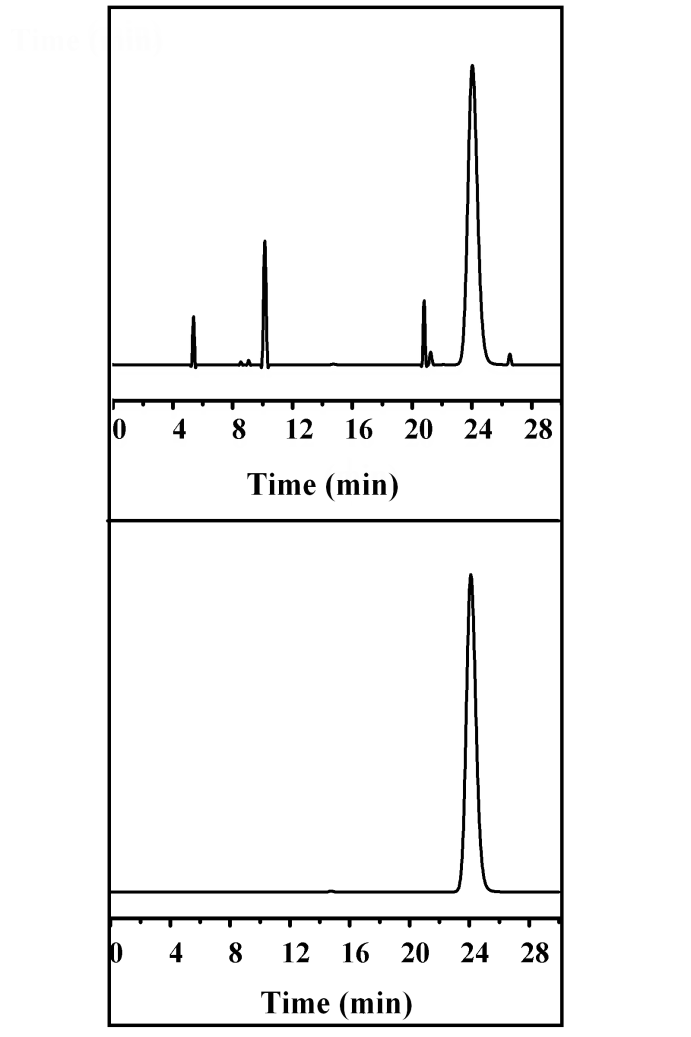

Supplement: Supplementary file 1 — Additional file 1. Additional figures. [file 13068_2020_1725_MOESM1_ESM.docx]
