# Supplementary material for: Biosynthesis of acetylacetone inspired by its biodegradation
Source: Biotechnol Biofuels. 2020 May 15;13:88. doi: 10.1186/s13068-020-01725-9 (PMC7226712; doi:10.1186/s13068-020-01725-9)
Supplement: Supplementary file 2 — Additional file 2. Additional tables. [file 13068_2020_1725_MOESM2_ESM.docx]

**Fed-batch fermentation**

During the fermentation, sterilized air was supplied at 1 vvm and ammonia was added automatically to control the pH 7. The agitation speed was set at 400 rpm and then associated with the dissolved oxygen to maintain the concentration at 20% saturation. The stirring rate was automatically adjusted from 400 to 800 rpm during the whole fermentation. Fed-batch mode was commenced by feeding 60% glucose when the dissolved oxygen increased. The glucose feeding rate was controlled at a range of 0-2 mL/min, the real-time rate was adjusted based on the residual glucose of the fermentation broth, and the residual glucose was kept at a range of 10-20 g/L. When the cell density reached to an OD_600_ of 30, the recombinant proteins were induced by 0.2 mM IPTG along with 0.5 mM FeSO_4_•7H_2_O added, and nitrogen was used for anaerobic conditioning after induction. The nitrogen was supplied at a flow rate of 2 L/min after induction to ensure oxygen-free environment. The temperature was adjusted to 30℃ for further cultivation. The agitation speed was kept at a constant rate of 200 rpm during anaerobic fermentation. 5 ml of the fermentation broth was withdrawn periodically to determine the cell density, residual glucose and product titer. The fed-batch fermentation was performed in triplicate. The DO-stir rate profile for the full fermentation was presented in Fig. S5.

**Standard curve establishment**

A stock standard (10 mg/mL) was prepared by dissolving 100 mg of acetylacetone in 10 mL of double distilled water. Calibration standards (100, 50, 25, 10, 5, 1, and 0.1 mg/L) were prepared by serial dilution of the stock standard. The calibration standards were analyzed by HPLC, and the retention time of acetylacetone was 24.1 min. The standard curve of y = 17.148 x + 6.267 (R^2^ = 0.9996) was established based on the standards concentration and the corresponding peak area. The representative chromatogram of the standard and the sample was shown in Fig. S6.

**Table S1** Information of proteins given >60% sequence identity with Dke1.

| NO. | protein name | Accession | ident | aa_length | species |
| --- | --- | --- | --- | --- | --- |
| Dke1 | Acetylacetone-cleaving  enzyme | Q8GNT2.1 | 100 | 153 | *Acinetobacter johnsonii* |
| model_1 | hypothetical protein | WP_062761874.1 | 71.724 | 151 | *Tistrella mobilis* |
| model_2 | acetylacetone-cleaving  protein | WP_014753275.1 | 71.034 | 151 | *Tistrella mobilis* |
| model_3 | acetylacetone-cleaving  protein | WP_095620360.1 | 69.388 | 148 | *Halomonas* sp. WRN001 |
| model_4 | acetylacetone-cleaving  protein | OYT99227.1 | 67.857 | 141 | *Burkholderiales bacterium*  PBB1 |
| model_5 | acetylacetone-cleaving  protein | WP_066254197.1 | 67.857 | 141 | *Hydrogenophaga flava* |
| model_6 | acetylacetone-cleaving  protein | WP_076204322.1 | 67.606 | 144 | *Rhodoferax koreense* |
| model_7 | acetylacetone-cleaving  protein | WP_077334430.1 | 67.143 | 141 | *Hydrogenophaga* sp. A37 |
| model_8 | acetylacetone-cleaving  protein | PKO64410.1 | 67.143 | 141 | *Betaproteobacteria*  *bacterium*  HGW-Betaproteobacteria-16 |
| model_9 | hypothetical protein | WP_011796996.1 | 66.901 | 145 | *Acidovorax citrulli* |
| model_10 | acetylacetone-cleaving  protein | WP_127765244.1 | 66.667 | 142 | *Rhodospirillaceae bacterium*  Gri0909 |
| model_11 | acetylacetone-cleaving  protein | RYF37058.1 | 65.734 | 145 | *Comamonadaceae*  *bacterium* |
| model_12 | acetylacetone-cleaving  protein | RZL68727.1 | 65.734 | 145 | *Variovorax* sp. |
| model_13 | acetylacetone-cleaving  protein | OYW20166.1 | 65.493 | 144 | *Burkholderiales bacterium*  12-64-5 |
| model_14 | acetylacetone-cleaving  protein | WP_081269232.1 | 65.035 | 144 | *Variovorax paradoxus* |
| model_15 | acetylacetone-cleaving  protein | WP_011831232.1 | 64.789 | 144 | *Methylibium* |
| model_16 | hypothetical protein | WP_056318988.1 | 64.789 | 144 | *Methylibium* sp. Root1272 |
|  |  |  |  |  |  |
| model_17 | hypothetical protein | WP_008373031.1 | 64.384 | 147 | *Pseudomonas* sp. M47T1 |
| model_18 | Acetylacetone  Dioxygenase | 2O1Q_A | 63.38 | 145 | *Methylibium Petroleiphilum*  Pm1 |
| model_19 | acetylacetone-cleaving  protein | WP_128870485.1 | 63.014 | 149 | *Pseudomonas* sp. VI4.1 |
| model_20 | acetylacetone-cleaving  protein | WP_086437557.1 | 62.406 | 145 | *Altererythrobacter*  *xiamenensis* |
| model_21 | acetylacetone-cleaving  protein | OUX50611.1 | 62.143 | 154 | *Methylococcaceae*  *bacterium* TMED282 |
| model_22 | acetylacetone-cleaving  protein | KUO54982.1 | 61.905 | 147 | *Alphaproteobacteria*  *bacterium* BRH_c36 |
| model_23 | acetylacetone-cleaving  protein | KTC27638.1 | 61.644 | 147 | *Pseudomonas* sp. ABAC61 |
| model_24 | acetylacetone-cleaving  protein | WP_066123161.1 | 60.39 | 155 | *Xylophilus ampelinus* |
| model_25 | acetylacetone-cleaving  protein | WP_119587158.1 | 60.15 | 145 | *Erythrobacter* sp. V18 |
| model_26 | hypothetical protein | WP_022975330.1 | 60.135 | 149 | *Nevskia ramosa* |
| model_27 | hypothetical protein | WP_007182343.1 | 60 | 143 | *Burkholderia* sp. Ch1-1 |

**Table S2** Primers used in this study for plasmids construction or allele verification.

| Primers | Description |
| --- | --- |
| pETDuet-up1_F | GATGCGTCCGGCGTAGAGC |
| T7-term_R | GCTAGTTATTGCTCAGCGG |
| Ajo_Dke1-K15Q_F | AAGAATACGTTCAAATCTCTGACAAAA |
| Ajo_Dke1-K15Q_R | TTGTTGTCAGAGATTTGAACGTATTCTT |
| Ajo_Dke1-S17D_F | GAAGAATACGTTAAAATCGATGACAACAACTACGTTCCGTTC |
| Ajo_Dke1-S17D_R | GAACGGAACGTAGTTGTTGTCATCGATTTTAACGTATTCTTC |
| Ajo_Dke1-Y21W_F | ATCTCTGACAACAACTGGGTTCCGTTCCCGGAAGCGT |
| Ajo_Dke1-Y21W_R | ACGCTTCCGGGAAGGAACCCAGTTGTTGTCAGAGAT |
| Ajo_Dke1-L103R_F | TTTCGATCTTCTGGTGCTCGGCACGGTAAAACC |
| Ajo_Dke1-L103R_R | GGTTTTACCGTGCCGAGCACCAGAAGATTCGAAA |
| Ajo_Dke1-G105D_F | TTCGAATCTTCTGGTGCTCTGCACGATAAAACCTTCTTCCCGGTTGAATC |
| Ajo_Dke1-G105D_R | GATTCAACCGGGAAGAAGGTTTTATCGTGCAGAGCACCAGAAGATTCGAA |
| Ajo_Dke1-A60N_F | GGCTGGTTCTTCTTTCAATTCTCACATCCACGCTG |
| Ajo_Dke1-A60N_R | CAGCGTGGATGTGAGAATTGAAAGAAGAACCAGCC |
| Ajo_Dke1-A60D_F | GGCTGGTTCTTCTTTCGATTCTCACATCCACGCTG |
| Ajo_Dke1-A60D_R | CAGCGTGGATGTGAGAATCGAAAGAAGAACCAGCC |
| Ajo_Dke1-G101N_F | ACGGTTTCGAATCTTCTAATGCTCTGCACGGTAAAAC |
| Ajo_Dke1-G101N_R | GTTTTACCGTGCAGAGCATTAGAAGATTCCAAACCGT |
| Ajo_Dke1-G101S_F | ACGGTTTCGAATCTTCTAGTGCTCTGCACGGTAAAAC |
| Ajo_Dke1-G101S_R | GTTTTACCGTGCAGAGCACTAGAAGATTCCAAACCGT |
| Ajo_Dke1-G101D_F | ACGGTTTCGAATCTTCTGATGCTCTGCACGGTAAAAC |
| Ajo_Dke1-G101D_R | GTTTTACCGTGCAGAGCATCAGAAGATTCCAAACCGT |
| Ajo_Dke1-L103C_F | TTCGAATCTTCTGGTGCTTGTCACGGTAAAACCTTC |
| Ajo_Dke1-L103C_R | GAAGGTTTTACCGTGACAAGCACCAGAAGATTCGAA |
| Ajo_Dke1-L103Q_F | TTCGAATCTTCTGGTGCTCAGCACGGTAAAACCTTC |
| Ajo_Dke1-L103Q_R | GAAGGTTTTACCGTGCTGAGCACCAGAAGATTCGAA |
| Ajo_Dke1-E140V_F | TTCTATCGGTTGGGCTGTAGCTCAGGGTGCTTGGCTGGCTACC |
| Ajo_Dke1-E140V_R | GGTAGCCAGCCAAGCACCCTCAGCTACAGCCCAATTGATAGAA |
| Ajo_Dke1_F_BamHI | CGGGATCCGATGGACTACTGCAACA |
| Ajo_Dke1_R_EcoRI | CGGAATTCTTAAGCAGCTTCGTTTTTGGTA |

**Table S3** The detailed information of HPLC.

| Instrument | Waters 1525 |
| --- | --- |
| Chromatographic column | 300 mm×7.8 mm Aminex HPX-87H (Bio-Rad, USA) |
| Detector | Varian Cary 50 UV-Vis, US |
| Wave length | 280 nm |
| Mobile phase | 5 mM H_2_SO_4_ |
| Flow rate | 0.6 mL/min |
| Column temperature | 60 ℃ |
| Injection volume | 10 μL |
| Running time | 30 min |
